# Green Credit Policy and Corporate Stock Price Crash Risk: Evidence From China

**DOI:** 10.3389/fpsyg.2022.891284

**Published:** 2022-04-25

**Authors:** Wei Zhang, Yun Liu, Fengyun Zhang, Huan Dou

**Affiliations:** ^1^School of Statistics, Shandong University of Finance and Economics, Jinan, China; ^2^School of Management, Jinan University, Guangzhou, China

**Keywords:** green credit policy, stock price crash risk, financial constraints, information transparency, corporate sustainability

## Abstract

Using the promulgation of *Green Credit Guidelines* in China as the research setting, this paper exploits a quasi-natural experiment to examine the impact of green credit policy on the stock price crash risk of heavy-polluting firms. The results show that green credit policy significantly increases the risk of stock price crash of heavy-polluting firms. Such impact is transmitted through increased financial constraints and reduced information transparency. In addition, we find that the impact of green credit policy on the stock price crash risk is more pronounced in firms with weak external governance and a small size. Our findings provide policy implications for mitigating corporate risks and promoting corporate sustainability.

## Introduction

The global sustainable development goals emphasize the urgency of environmental governance (Clark, 2018; Sun et al., 2019). Environmental regulation has become a common means for balancing economic and environmental development. As a vital aspect of environmental regulation, green credit policy (GCP) has a greater influence than other green financial policies such as green bonds, green insurance, and green crowdfunding policy (Wu and Yin, 2021). The definition of “green credit” is based on the Equator Principles established in 2002 by the International Finance Corporation (IFC) and ABN AMRO Bank, which, although not legally binding, has become a new standard in international project financing. Green credit is gaining popularity as a tool for environmental protection and corporate development. By 2020, 113 financial institutions across 37 countries have recognized the Equator Principles. In China, the green credit policy has also been refined: in 2007, the *Opinions on Implementing Environmental Protection Policies and Regulations to Prevent Credit Risks* included green credit policies for the first time, and in 2012, the *Green Credit Guidelines* laid out more specific and detailed requirements (Zhang et al., 2011; Wang et al., 2019). By 2021, China’s balance of green credit has grown to RMB159,000 yuan, according to the People’s Bank of China. GCP involves a set of policies and institutional arrangements aimed at restricting the flow of credit to heavy-polluting firms while increasing the flow of credit to non-heavy-polluting firms that participate in environmental and energy-saving projects through loan products, loan terms, loan interest rates and loan limits, which ultimately influences firms’ environmental behaviors (Nandy and Lodh, 2012; Wen et al., 2021). Prior research shows that GCP incentivizes heavy-polluting firms to innovate and reform (Wang and Wang, 2021), as well as improve productivity (Zhang, 2021) and resource allocation (Zhou et al., 2021). From the perspective of corporate behavior, Liu et al. (2021) find that “green” commitment lowers a firm’s stock price crash risk. In terms of green credit regulation, Fan et al. (2021) discover that GCP has a significantly positive impact on the loan interest rates, loan size and financing costs of non-heavy-polluting firms, but a significantly negative impact on heavy-polluting firms. However, there is no systematic analysis on the relationship between GCP and stock price crash risk in the existing literature. Research on this issue has important implications for mitigating the financial risks of capital markets and maintaining stock market stability.

Stock price crashes damage a firm’s value and cause loss of wealth for investors. Corporate sustainability and stock market stability will also be jeopardized. Since the outbreak of the global financial crisis in 2008, “stock price crash risk” has been one of the focal points of academic discussions (Zhang et al., 2021; Kang et al., 2022; Tian et al., 2022a). On the one hand, principal-agent and information asymmetry theories suggest that stock price crashes occur as a result of managers concealing bad news about the firm. This behavior leads to information asymmetry between investors and the firm. On the other hand, with the implementation of the GCP, the risk of loan default of heavy-polluting firms has increased, forcing banks and other financial institutions to cut loan sizes and tighten financing constraints for these firms to mitigate the risk. The tightened financing constraints thus increases the stock price crash risks of heavy-polluting firms. Therefore, we believe that GCP influences stock price crash risk through two channels: corporate financial constraints, which can be measured by corporate loan size, corporate loan cost, and the *SA* index (Hadlock and Pierce, 2010; Yao et al., 2021), and information transparency, which can be measured by common financial reporting quality indicators like earnings quality (*DD*), corporate disclosure score (*DSCORE*), number of analysts following (*ANALYST*) and analysts’ earnings forecast accuracy (*ACCURACY*) (Graham et al., 2005; Huddart et al., 2009).

Information asymmetry or opacity is one of the main causes of stock price crashes (Gaspar et al., 2005; Piotroski et al., 2015). The key factors influencing corporate information transparency include conflicts of interest between managers and shareholders (Jin and Myers, 2006; Kim et al., 2011a,b), media coverage quality (Fang and Peress, 2009; Zou et al., 2019), and audit quality (Bleck and Liu, 2007). For self-serving purposes, managers may selectively disclose information about the firm and conceal bad news, limiting information transparency and making it harder for outside investors to assess genuine corporate performance in a timely manner. When bad news builds up to a particular point before being revealed to the market, it can be a fatal blow to the firm’s stock performance and potentially lead to a stock price crash (Jin and Myers, 2006; Hutton et al., 2009; Kim and Zhang, 2016). Meanwhile, GCP has a negative impact on the information transparency of heavy-polluting firms, which in turn increases their risk of price crashes. As GCP severely restricts the flow of credit to heavy-polluting firms, these firms are more prone to conceal bad news or obfuscate information in order to circumvent the policy restrictions (Eiler et al., 2015; Wang et al., 2015; He et al., 2019).

China provides the ideal research setting for exploring the impact of GCP on stock price crash risk (Su et al., 2020; Wan et al., 2021). On the one hand, it is the world’s largest carbon emitter, and environmental issues have become a major concern for both the government and the general public (Xue et al., 2021; Liu et al., 2022; Tian et al., 2022b; Zhai et al., 2022). In recent years, China has taken a number of environmental initiatives, including a target to peak CO_2_ emissions by 2030 and achieve carbon neutrality by 2060, which was declared during the 75th UN General Assembly in 2020. On the other hand, the introduction of *Green Credit Guidelines* in 2012 is an exogenous and firm-independent event for Chinese firms, which provide an ideal context for a quasi-natural experiment. Using China as the research setting is consistent with our research hypotheses. Finally, the escalating environmental issues in China resulting from its rapid economic development catalyzed the promulgation of green credit policy. Therefore, the policy aims to reform the high energy-consuming, high-polluting economic structure, making it highly relevant to our research.

Using Chinese A-share listed firms^1^ as the research setting, this paper empirically examines the impact of GCP on stock price crash risk by employing the difference-in-differences (DID) model to measure stock price volatility before and after the adoption of GCP. The results show that, first, GCP increases the risk of heavy-polluting firms experiencing price crashes, and this finding is robust. Second, the heterogeneity analysis suggests that GCP has a more pronounced impact on the stock price crash risk of heavy-polluting firms with poor external governance and a small size. Finally, the mediating analysis reveals that corporate financial constraints and information transparency play a substantial role in mediating the relationship between GCP and the stock price crash risk of heavy-polluting firms. By examining whether and how GCP affects stock price crash risk, our paper provides a reference for mitigating corporate risks, improving corporate sustainability, and developing a cohesive macro-financial policy framework.

The remainder of this paper is organized as follows: section “Theoretical Background and Hypotheses Development” provides the theoretical background and hypothesis development; section “Research Design” introduces the research design; section “The Impact of Green Credit Policy on Stock Price Crash Risk” explains the baseline results; section “Robustness Checks” presents the robustness checks; section “Heterogeneity Analysis” is the heterogeneity analysis; section “Mediating Analysis” provides the mediating analysis; section “Conclusions and Policy Implications” concludes the paper.

## Theoretical Background and Hypotheses Development

### Institutional Background

**FIGURE 1:**
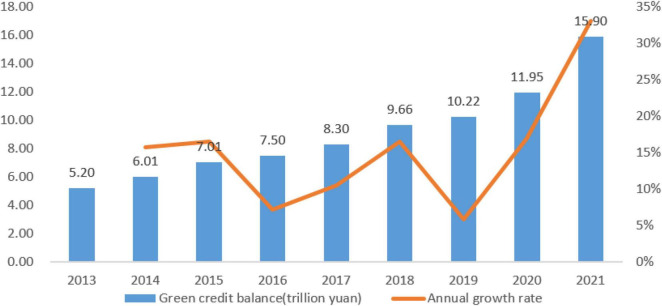
Balance and annual growth rate of green credit in domestic and foreign currencies of major financial institutions in China, 2013–2021.

To address environmental challenges, the Chinese government has prioritized environmental governance and implemented a number of initiatives and policies.^2^ However, the lack of an effective monitoring and regulatory mechanism has resulted in a limited effect of these policies on raising environmental awareness among businesses and a significant gap between intended and achieved goals (Tian et al., 2020; Yang et al., 2020). According to the *2019 Bulletin of Ecology and Environment Status of China*, 53.4% of Chinese cities exceeded ambient air quality standards in 2019, with 337 cities experiencing 452 days of severe pollution, an increase of 88 days from 2018.^3^ As a result, “market” mechanism is still needed in addressing environmental problems. Green credit policy (GCP) incorporates environmental governance in corporate development through a differentiated loan granting policy (Zhong et al., 2021; Shi et al., 2022). In 2007, the Environmental Protection Administration, People’s Bank of China, and China Banking Regulatory Commission (CBRC) jointly issued the *Opinions on Implementing Environmental Protection Policies and Regulations to Prevent Credit Risks* to curb the blind expansion of high energy-consuming and polluting industries. It requires commercial banks to undertake credit control over firms and projects that do not comply with industrial policies and environmental objectives and make environmental performance a condition for loan granting. In 2012, the CBRC issued the *Green Credit Guidelines*, which supplemented the *Opinions* and improved the GCP’s operability. According to the People’s Bank of China, the green credit balance in China’s domestic and foreign currencies was 5.20 trillion yuan in 2013 and would expand to 15.9 trillion yuan in 2021, with an average annual growth rate of 15%.^4^ Since the inception of the GCP, the scale of green credit has been on the rise year by year (as shown in Figure 1).

### Hypotheses Development

Stock price crash risk refers to the probability of stock prices falling swiftly and sharply as a result of a large number of investors selling stocks for a specific reason (Hong et al., 2017). Stocks are notoriously volatile, with price crashes occurring more frequently than price surges (Bekaert and Wu, 2000). In terms of the impact sphere, stock price crashes can be divided into market-level and firm-level stock price crashes. The 2007 U.S. financial crisis and the 2015–2016 China stock market crash are two well-known examples of the market-level stock price crash. Meanwhile, typical examples of the firm-level crashes include (1) the “D’long incident” in 2004, in which the D’long International Strategic Investment Company blindly expanded in the absence of funding, eventually leading to the collapse of its share price; and (2) the stock price crash of Huishan Dairy in 2018, which occurred in less than half an hour owing to the exaggerated profits and profitability being exposed. The stock price crash severely infringes on the interests of stakeholders and has serious economic ramifications.

Research on stock price crash risk primarily focuses on its formation mechanism, measurement method, and influencing factors. The most prominent topic among them is influencing factors, and most of the existing literature on this topic concentrates on internal and external corporate governance. From the perspective of internal corporate governance, separation of corporate ownership and management led by refinement of social division of labor has resulted in a principal-agent problem. The principal-agent theory suggests that business owners expect managers to maximize resource allocation and economic benefits while maintaining strong environmental performance and a positive firm image to be able to obtain green credit and government subsidies. Simultaneously, corporate managers seek to avoid negative evaluations led by the exposure of environmental problems such as severe pollution by focusing on green innovation and resource conservation. However, managers may act short-sightedly in pursuit of self-serving interests. They tend to conceal or delay the disclosure of bad news owing to various concerns related to career prospect (Kothari et al., 2009), colleague respect (Ball, 2009), promotion (Piotroski et al., 2015), and equity (Kim et al., 2011b). Information asymmetry theory suggests that managers may use their information advantage to engage in short-sighted conduct that damages environmental causes, corporate development, and the interests of the information disadvantaged parties such as investors. Graham et al. (2005) find that managers are more likely to delay or conceal bad news when confronted with it. The concealing of substantial amounts of unfavorable news results in low business transparency and significant degrees of information asymmetry. The exposure of such news could lead to a stock price crash (Benmelech et al., 2010). Thus, the more transparent a firm’s information disclosure, the lower its stock price crash risk (Chen et al., 2001; Kim and Zhang, 2016). Therefore, information transparency helps to reduce information asymmetry and a firm’s risk of price crashes.

With the adoption of the GCP, heavy polluters may face huge environmental liability as a result of pollution control, and financial institutions will lower their loan size to hedge the risks. Some studies have shown that when firms have information asymmetry with investors, low information transparency, and high default risk, they are unable to obtain long-term debt financing (Goss and Roberts, 2011; Ge and Liu, 2015). In addition, the regulatory constraints increase the risk of heavy-polluting firms defaulting on their loans owing to corporate infractions (Cai et al., 2019). Moreover, once negative news of heavy-polluting firms like major environmental pollution was exposed, banks are likely to reduce their loan size and increase their financing constraints under public pressure.

Under the GCP, heavy-polluting firms are reluctant to disclose environmental information in order to circumvent the policy restrictions, while firms with good environmental performance are more willing to disclose environmental information (Peters and Romi, 2013). This is because, in order to minimize debt costs, financial institutions seek to subsidize firms that provide more environmental information (Eliwa et al., 2021). According to Chen et al. (2014), the more transparent a firm’s corporate disclosure is, the more detailed the environmental information it discloses. Both the financing ability and information transparency of heavy-polluting firms are closely related to their share price crash risk. Therefore, we propose the first hypothesis.

Hypothesis 1: Green credit policy increases the stock price crash risk of heavy-polluting firms.

As an important mechanism affecting the cost of corporate financing and information transparency, external corporate governance significantly affects the risk of stock price crash. External corporate governance refers to the mechanism through which outsiders, such as auditors, institutional investors, media, and securities analysts, monitor and supervise the acts of a firm’s controlling shareholders and decision-makers such as managers and boards of directors (Bradshaw, 2011; Mayew et al., 2013; Solomon and Soltes, 2015; Gao et al., 2017). First, reputation theory suggests that external regulators, such as auditors and securities analysts, have the ability and motivation to detect and disclose corporate violations in a timely manner. Their in-depth knowledge of a firm’s overall performance allows them to uncover corporate infractions sooner than ordinary investors. Also, it is one of their responsibilities to protect investors from investment losses in firms with irregularities, and the prompt disclosure of corporate irregularities enhances their reputation and career prospects (Yuan and Zhu, 2022).

Second, in firms with poor external corporate governance, management may override corporate governance mechanisms, which would increase the occurrence of managers’ self-interested behavior. Adams et al. (2005) and Cheng (2008) find that the greater the power of managers, the more volatile corporate performance becomes. Also, Quan et al. (2010) find that when managers have more power, they are more likely to engage in earnings management for higher pay, which would directly damage the firm’s value. In China’s institutional setting, the level of corporate information transparency has a strong correlation with managerial conduct. Kothari et al. (2009) find that managers tend to conceal or delay the disclosure of bad news when it happens, leading to information opacity. This is especially true for heavy-polluting firms since the implementation of the GCP, as their information transparency has significantly decreased (Wang et al., 2015). However, a more transparent information disclosure can reduce the stock price crash risk and enhance market liquidity (Bloomfield and Wilks, 2000; Patel and Dallas, 2003). Hence, we propose the second hypothesis.

Hypothesis 2: Green credit policy has a more significant impact on the stock price crash risk of firms with poor external corporate governance.

Prior research shows that the extent of financial constraints imposed by banks on a firm is proportional to the size of the firm. First, the loan repayment capacity and business risks of borrowing firms are important factors for banks and other financial institutions to consider. Banks are more inclined to lend money to large firms because their assets can be used to repay the debt even if they go bankrupt and liquidate. Tong (2011) argues that larger firms own more capital, social influence, and resource integration capabilities than smaller firms. They also have a larger pool of potential collateral assets, a better reputation and business credit, and fewer financial constraints. Moreover, larger firms are better at risk-diversification, especially in the event of an exogenous occurrence, such as the introduction of GCP. Furthermore, large firms have a broader and more diverse group of stakeholders (Branco and Rodrigues, 2008), comprising trading partners like shareholders, creditors, employees, consumers, and suppliers, as well as supervisory bodies like the government and the media (Li and Zhang, 2020; Dumitrescu and Zakriya, 2021). These stakeholders have direct or indirect influence with the production and operation activities of firms. They share certain business risks with the firms while monitoring and controlling their production and operation activities. On the one hand, stakeholders require firms to actively disclose information in order to understand the real condition of firm operation and reduce information asymmetry. On the other hand, stakeholders increase their monitoring and supervision of firms to avoid economic losses potentially caused by the self-serving conduct of firm managers. As such, large firms have a higher level of information transparency. Therefore, after the introduction of GCP, heavy-polluting firms with a large size will have lower financing constraints, higher information transparency, and lower risk of stock price crash than heavy-polluting firms with a smaller size. Hence, we propose the third hypothesis.

Hypothesis 3: Green credit policy has a greater influence on the stock price crash risk of small firms than that of large firms.

## Research Design

### Model Specification

Based on the difference-in-difference (DID) model, we construct Model (1) to examine the impact of green credit policy (GCP) on stock price crash risk.


$$C\,r\,a\,s\,h\,r\,i\,s\,k_{i\,t+1}=\alpha +\beta_{1}\,P\,o\,l\,i\,c\,y_{t}\times t\,r\,e\,a\,t_{i}+\beta_{2}\,t\,r\,e\,a\,t_{i}$$

(1) $$+\beta_{3}\,P\,o\,l\,i\,c\,y_{t}+\gamma^{\prime }\,x_{i\,t}+\varepsilon_{i\,t}$$

Following (Kim et al., 2011a,b), we use the skewness of negative stock return (*NCSKEW*) and the fluctuations in stock return (*DUVOL*) to measure stock price crash risk. In Model (1), *Crashrisk* is the stock price crash risk as measured by *NCSKEW* and *DUVOL*. *Policy* indicates the implementation of GCP; following (Hu et al., 2021; Yao et al., 2021), it is set to the value of 1 for the years after 2012 when the *Green Credit Guidelines* was issued, and 0 otherwise. *Treat* denotes whether the firm is in a heavy-polluting industry or whether it is subject to financial constraints imposed by the GCP; it is set to the value of 1 when the firm belongs to Category A industry or is a heavy-polluting firm (treatment group), and 0 when the firm is non-heavy-polluting firm (control group). *Policy* × *treat* is the interaction term of GCP and heavy-non-heavy- polluting firms; it measures the impact of the GCP on the stock price crash risk of heavy-polluting and non-heavy-polluting firms. β_*1*_ reflects the impact of GCP on the stock price crash risk of heavy-polluting and non-heavy-polluting firms before and after its implementation. If β_*1*_ is significantly greater than 0, it means that GCP significantly contributes to the stock price crash risk of heavy-polluting firms; otherwise, there is no significant contribution.

Based on prior studies (Chen et al., 2001; Kim et al., 2014), we also control for the following factors to eliminate their impacts on stock price crash risk: leverage ratio (*Lev*_*t*_), profitability (*ROE*_*t*_), net operating cash flow (*Cashflow*_*t*_), equity balance (*Balance*_*t*_), monthly stock turnover rate (*Dturn*_*t*_), firm age (*age*_*t*_), firm size (*lnsize*_*t*_), *TobinQ*_*t*_, the standard deviation of weekly stock return (*Sigma*_*t*_), mean weekly stock return (*Ret*_*t*_). These control variables are denoted by *x*_*it*_. Detailed descriptions of all variables are provided in Appendix Table 1. To eliminate the effects of unobservable factors and heteroscedasticity, we adopt the dual-cluster model that controls for time fixed effects and industry fixed effects, and robust standard errors are clustered at the industry level.

Table 1 provides the summary statistics. The variables of *NCSKEW* and *DUVOL* have a mean value of −0.2417 and −0.1834 and a standard deviation of 0.9144 and 0.7435, respectively. This indicates that the two variables differ significantly among the sample firms, and the statistical results are similar to those of Chen et al. (2001) and Xu et al. (2012).

**TABLE 1:** Summary statistics.

Variable	N	Mean	Std.err	5%	25%	Median	75%	95%
*NCSKEW*_*t+1*_	6,594	−0.2417	0.9144	−1.7369	−0.7812	−0.2517	0.3137	1.2474
*DUVOL*_*t+1*_	6,594	−0.1834	0.7435	−1.4152	−0.6580	−0.2076	0.2743	1.1257
*Lev*_*t*_	6,594	0.5213	0.1825	0.2051	0.3871	0.5259	0.6622	0.8084
*ROE*_*t*_	6,594	0.0757	0.1040	−0.0796	0.0287	0.0731	0.1240	0.2376
*Cashflow*_*t*_	6,594	0.0428	0.0710	−0.0789	0.0044	0.0427	0.0841	0.1608
*Balance*_*t*_	6,594	0.5332	0.5163	0.0414	0.1354	0.3683	0.7675	1.6258
*Dturn*_*t*_	6,594	0.0413	0.3147	−0.4772	−0.1254	0.0227	0.2078	0.6001
*age*_*t*_	6,594	2,001	5.3144	1,993	1,996	2,000	2,004	2,010
*lnsize*_*t*_	6,594	3.1065	0.0540	3.0268	3.0691	3.0994	3.1397	3.2067
*Sigma*_*t*_	6,594	0.0500	0.0182	0.0253	0.0373	0.0474	0.0593	0.0865
*Ret*_*t*_	6,594	0.0474	4.2081	−6.4174	−2.1556	−0.4336	1.9267	7.9269
*TobinQ*_*t*_	6,594	1.9621	1.2212	1.0026	1.2294	1.5896	2.2502	4.1938
*policy*_*t*_	6,594	0.6794	0.4667	0	0	1	1	1
*treat*_*i*_	6,594	0.0855	0.2797	0	0	0	0	1
*treat*_*i*_×*policy*_*t*_	6,594	0.0604	0.2382	0	0	0	0	1

### Sample and Data Source

In 2012, the China Banking Regulatory Commission (CBRC) established the *Green Credit Guidelines*, dividing industries into categories A, B, and C based on environmental and social risk as defined in the *Key Performance Indicators for Implementing Green Credit*. Category A includes industries whose construction, production, and operation activities are likely to seriously change the original ecology and produce adverse environmental and social consequences.^5^ Category B includes industries whose construction, production and operation activities may produce adverse environmental and social consequences but can be easily mitigated; Category C includes industries that do not have an adverse impact on the environment. Using Chinese A-share listed firms as the research setting, we set the sample interval as the 3 years before and after 2012 (the year of the adoption of the *Green Credit Guidelines*), namely, 2009–2015. The sample was screened according to the following criteria: (1) excluding firms in the financial and insurance industries; (2) excluding ST, ST*, and PT firms; (3) excluding firms with asset-liability ratios less than 0 and greater than 1; (4) excluding firms with missing values; (5) excluding extreme values after all continuous variables are winsorized at the 1 and 99% levels. Eventually, 6,594 observations were obtained. The data on institutional investors’ holdings used in this paper comes from the Wind database,^6^ and other data comes from the *China Stock Market Accounting Research* (CSMAR).^7^

## The Impact of Green Credit Policy on Stock Price Crash Risk

### Baseline Results

Table 2 reports the baseline results. Columns (1) and (2) show the regression results without control variables, whereas columns (3) and (4) show the regression results with control variables. As shown in columns (1) and (2), the coefficients of *treat*_*i*_×*policy*_*t*_ are 0.1277 and 0.1639 and significantly positive at the 5 and 1% level, respectively. This indicates that the stock price crash risk of heavy-polluting firms has increased significantly after the implementation of the green credit policy (GCP). The results in columns (3) and (4) show that after controlling for other factors that may affect stock price crash risk, the coefficients of *treat*_*i*_×*policy*_*t*_ increase to 0.1534 and 0.1929 and are significantly positive at the 5 and 1% level, indicating that the implementation of the GCP makes the stock price crash risk significantly higher for heavy-polluting firms. The findings prove Hypothesis 1.

**TABLE 2:** Baseline regression results.

	(1)	(2)	(3)	(4)
Variable	*NCSKEW*_*t+1*_	*DUVOL*_*t+1*_	*NCSKEW*_*t+1*_	*DUVOL*_*t+1*_
*treat*_*i*_×*policy*_*t*_	0.1277**	0.1639***	0.1534**	0.1929***
	(2.0719)	(3.2871)	(2.4195)	(4.1010)
*policy*_*t*_	0.0130	0.1129**	−0.0589	0.0320
	(0.2406)	(2.2964)	(−0.9586)	(0.5701)
*treat*_*i*_	−0.1256***	−0.1199***	−0.1354***	−0.1162***
	(−3.0729)	(−3.5462)	(−3.0248)	(−3.8209)
*Lev*_*t*_			−0.0834	−0.1564**
			(−0.9741)	(−2.6433)
*ROE*_*t*_			0.3794***	0.1501
			(2.8683)	(1.5753)
*Cashflow*_*t*_			−0.3159	−0.2403
			(−1.4731)	(−1.4092)
*Balance*_*t*_			0.0090	−0.0001
			(0.4332)	(−0.0071)
*Dturn*_*t*_			−0.0845	−0.0943**
			(−1.5419)	(−2.0683)
*age*_*t*_			0.0005	0.0014
			(0.1482)	(0.5554)
*lnsize*_*t*_			−0.4172	0.1978
			(−1.0958)	(0.7600)
*NCSKEW*_*t*_			0.0230*	
			(1.8378)	
*Sigma*_*t*_			3.9500***	3.2817***
			(3.9328)	(4.0922)
*Ret*_*t*_			−0.0047	−0.0019
			(−1.3990)	(−0.6987)
*TobinQ*_*t*_			0.0111	0.0157*
			(0.8625)	(1.7315)
*DUVOL*_*t*_				−0.0190
				(−1.4163)
Constant	−0.1984***	−0.2969***	−0.0699	−3.7714
	(−3.3725)	(−5.3161)	(−0.0106)	(−0.7907)
*Industry fixed effects*	Yes	Yes	Yes	Yes
*Year fixed effects*	Yes	Yes	Yes	Yes
*Observations*	6,680	6,680	6,594	6,594
*R-squared*	0.0861	0.1112	0.0938	0.1182

(1) Robust standard errors are clustered by industry and t-statistics are reported in parentheses; (2) *, **, *** represent significant at the 10, 5, and 1% significance level, respectively.

## Robustness Checks

In this section, we will conduct a battery of robustness checks on the empirical results, including placebo tests, PSM-DID, alternative measures of dependent variables, expanding the heavy-polluting firm sample group, and adding CEO-level control variables.

### Placebo Test

To eliminate the influence of other factors on the stock price crash risk of heavy-polluting firms other than the green credit policy (GCP), we advance the implementation date of GCP by 1 year and 2 years, assuming that the policy was implemented in 2011 and 2010, respectively. We then re-estimate the baseline regression using the updated time, and the results are shown in Table 3. The results reveal that the coefficients of *treat*_*i*_×*beforepolicy*_1*t*_ and *treat*_*i*_×*beforepolicy*_2*t*_ are not significant, indicating that GCP has no effect on the stock price crash risk of heavy-polluting firms. This proves that the risk of a price crash for heavy-polluting firms is indeed influenced by the GCP rather than other factors.

**TABLE 3:** Placebo test.

	(1)	(2)	(3)	(4)
Variable	*NCSKEW*_*t+1*_	*DUVOL*_*t+1*_	*NCSKEW*_*t+1*_	*DUVOL*_*t+1*_
*treat*_*i*_×*beforepolicy*_1*t*_	0.1813	0.1824*		
	(1.5374)	(1.9223)		
*beforepolicy*_1*t*_	−0.0598	0.0348		
	(−0.9632)	(0.6068)		
*treat*_*i*_×*beforepolicy*_2*t*_			0.3394	0.2316
			(1.3337)	(0.9524)
*beforepolicy*_2*t*_			−0.0718	0.0317
			(−1.0925)	(0.5136)
*treat*_*i*_	−0.1726*	−0.1250	−0.3360	−0.1877
	(−1.7004)	(−1.5792)	(−1.3978)	(−0.8262)
*Lev*_*t*_	−0.0834	−0.1564**	−0.0823	−0.1557**
	(−0.9729)	(−2.6409)	(−0.9612)	(−2.6322)
*ROE*_*t*_	0.3786***	0.1495	0.3819***	0.1525
	(2.8896)	(1.5976)	(2.9279)	(1.6427)
*Cashflow*_*t*_	−0.3158	−0.2423	−0.3184	−0.2468
	(−1.4626)	(−1.4081)	(−1.4715)	(−1.4290)
*Balance*_*t*_	0.0090	0.0001	0.0092	0.0005
	(0.4388)	(0.0087)	(0.4466)	(0.0326)
*Dturn*_*t*_	−0.0863	−0.0963**	−0.0866	−0.0960**
	(−1.5896)	(−2.1275)	(−1.5700)	(−2.0937)
*age*_*t*_	0.0004	0.0013	0.0004	0.0012
	(0.1322)	(0.5062)	(0.1317)	(0.4799)
*lnsize*_*t*_	−0.4202	0.1924	−0.4201	0.1896
	(−1.1015)	(0.7334)	(−1.1019)	(0.7228)
*NCSKEW*_*t*_	0.0226*		0.0233*	
	(1.8208)		(1.8647)	
*Sigma*_*t*_	3.9296***	3.2494***	3.9554***	3.2542***
	(3.9156)	(4.0463)	(3.9458)	(4.0562)
*Ret*_*t*_	−0.0046	−0.0019	−0.0045	−0.0018
	(−1.3901)	(−0.6784)	(−1.3699)	(−0.6527)
*TobinQ*_*t*_	0.0109	0.0153*	0.0107	0.0151
	(0.8433)	(1.6798)	(0.8314)	(1.6528)
*DUVOL*_*t*_		−0.0193		−0.0188
		(−1.4442)		(−1.3976)
*Constant*	0.0484	−3.5159	0.0645	−3.3678
	(0.0073)	(−0.7324)	(0.0098)	(−0.7027)
*Industry fixed effects*	Yes	Yes	Yes	Yes
*Year fixed effects*	Yes	Yes	Yes	Yes
*Observations*	6,594	6,594	6,594	6,594
*R-squared*	0.0948	0.1179	0.0942	0.1178

(1) Robust standard errors are clustered by industry and t-statistics are reported in parentheses; (2) *, **, *** represent significant at the 10, 5, and 1% significance level, respectively.

### Propensity Score Matching-Difference-in-Differences

To mitigate potential endogeneity problems caused by sample selection bias, we adopt the propensity score matching (PSM) method for robustness check. In this paper, we use the kernel matching method with *Lev*_*t*_,*ROE*_*t*_,*Cashflow*_*t*_, *Balance*_*t*_,*Dturn*_*t*_, *age*_*t*_,*lnsize*_*t*_, *Sigma*_*t*_,*Ret*_*t*_ as covariates to match the treatment group, and the differences of covariates before and after matching are shown in Table 4. The propensity scores are estimated using a logit model. The matched sample size is 6,340. As shown in Table 4, the standardized bias (% bias) of all variables after matching is less than 10%, and none of the *t*-test results rejects the null hypothesis that there is no systematic difference between the treatment and control groups. The regression results remain robust as shown in columns (1) and (2) in Table 5.

**TABLE 4:** Matching results.

	Unmatched	Mean		% reduct		*t*-test		V(T)/V(C)
Variable	Matched	Treated	Control	% bias	|bias|	t	p > | t|	
*Lev*_*t*_	U	0.55	0.51	19.40		4.54	0.00	0.90
	M	0.55	0.55	2.20	88.60	0.40	0.69	0.94
*ROE*_*t*_	U	0.09	0.07	13.70		3.26	0.00	0.97
	M	0.09	0.08	4.10	69.80	0.71	0.48	0.87
*Cashflow*_*t*_	U	0.08	0.05	50.90		11.87	0.00	0.88
	M	0.08	0.07	6.60	87.10	1.12	0.26	0.79*
*Balance*_*t*_	U	0.52	0.54	−4.30		−1.09	0.28	1.28*
	M	0.52	0.53	−2.70	38.00	−0.47	0.64	1.29*
*Dturn*_*t*_	U	0.04	0.04	−0.20		−0.05	0.96	0.92
	M	0.04	0.04	−0.20	9.40	−0.04	0.97	1.25*
*age*_*t*_	U	2000	2001	−18.80		−4.15	0.00	0.66*
	M	2000	2000	−0.20	98.70	−0.04	0.96	0.74*
*lnsize*_*t*_	U	3.14	3.10	59.50		14.99	0.00	1.27*
	M	3.14	3.13	8.90	85.00	1.48	0.14	0.99
*Sigma*_*t*_	U	0.04	0.05	−37.90		−8.88	0.00	0.91
	M	0.04	0.04	−5.80	84.60	−1.07	0.29	1.05
*Ret*_*t*_	U	−0.10	0.08	−4.20		−0.99	0.32	0.90
	M	−0.10	−0.09	−0.20	94.60	−0.04	0.97	1.00

(1) If variance ratio outside (0.85; 1.17) for U and (0.85; 1.17) for M; (2) * represents significant at the 10% significance level.

**TABLE 5:** Robustness checks.

	(1)	(2)	(3)	(4)	(5)	(6)	(7)
Variable	*NCSKEW*_*t+1*_	*DUVOL*_*t+1*_	*CRASH*	*NCSKEW*_*t+1*_	*DUVOL*_*t+1*_	*NCSKEW*_*t+1*_	*DUVOL*_*t+1*_
*treat*_*i*_×*policy*_*t*_	0.1433**	0.1896***	0.0614***	0.1534**	0.1929***	0.1467**	0.1889***
	(2.4518)	(4.1240)	(2.8790)	(2.4195)	(4.1010)	(2.2803)	(4.0020)
*policy*_*t*_	−0.0665	0.0256	0.1654***	−0.0589	0.0320	−0.0571	0.0328
	(−1.0876)	(0.4444)	(5.8762)	(−0.9586)	(0.5701)	(−0.9043)	(0.5728)
*treat*_*i*_	−0.1410***	−0.1325***	−0.0258	−0.5380***	−0.3244***	−0.0978**	−0.0929***
	(−3.3808)	(−4.3101)	(−1.5450)	(−18.4063)	(−14.0536)	(−2.1577)	(−2.9720)
*Lev*_*t*_	−0.0590	−0.1459**	0.0210	−0.0834	−0.1564**	−0.0924	−0.1628***
	(−0.7007)	(−2.5744)	(0.9326)	(−0.9741)	(−2.6433)	(−1.0788)	(−2.7262)
*ROE*_*t*_	0.3659***	0.1269	−0.0685*	0.3794***	0.1501	0.3632**	0.1291
	(2.7268)	(1.2695)	(−1.9009)	(2.8683)	(1.5753)	(2.5464)	(1.3005)
*Cashflow*_*t*_	−0.3848	−0.2863	0.0422	−0.3159	−0.2403	−0.2811	−0.2083
	(−1.5608)	(−1.5361)	(0.8969)	(−1.4731)	(−1.4092)	(−1.2828)	(−1.2161)
*Balance*_*t*_	0.0076	−0.0010	0.0106*	0.0090	−0.0001	0.0072	−0.0024
	(0.3689)	(−0.0594)	(1.7249)	(0.4332)	(−0.0071)	(0.3575)	(−0.1496)
*Dturn*_*t*_	−0.0627	−0.0692	−0.0246*	−0.0845	−0.0943**	−0.0939*	−0.1042**
	(−1.0818)	(−1.4479)	(−1.8995)	(−1.5419)	(−2.0683)	(−1.6724)	(−2.2716)
*age*_*t*_	0.0007	0.0015	−0.0006	0.0005	0.0014	0.0013	0.0020
	(0.2294)	(0.6186)	(−0.8303)	(0.1482)	(0.5554)	(0.3715)	(0.7530)
*lnsize*_*t*_	−0.4125	0.2028	−0.2039**	−0.4172	0.1978	−0.4060	0.2037
	(−1.0660)	(0.7562)	(−2.5719)	(−1.0958)	(0.7600)	(−1.0559)	(0.7636)
*NCSKEW*	0.0249*			0.0230*		0.0213*	
	(1.9062)			(1.8378)		(1.7319)	
*Sigma*	4.2814***	3.3970***	0.0142	3.9500***	3.2817***	3.8400***	3.1249***
	(3.7712)	(3.7425)	(0.0534)	(3.9328)	(4.0922)	(3.8795)	(3.8981)
*Ret*_*t*_	−0.0045	−0.0018	−0.0004	−0.0047	−0.0019	−0.0049	−0.0023
	(−1.3109)	(−0.6384)	(−0.4834)	(−1.3990)	(−0.6987)	(−1.4375)	(−0.8288)
*TobinQ*_*t*_	0.0307**	0.0311***	−0.0041	0.0111	0.0157*	0.0141	0.0182**
	(2.1301)	(2.8990)	(−1.1685)	(0.8625)	(1.7315)	(1.0461)	(2.0096)
*IsDuality*_*i*_						−0.0320	−0.0133
						(−1.0032)	(−0.6091)
*gender*_*i*_						0.0152	−0.0083
						(0.3484)	(−0.2608)
*oveseaback*_*i*_						−0.0092	−0.0131
						(−0.1843)	(−0.3153)
*DUVOL*_*t*_		−0.0184			−0.0190		−0.0219
		(−1.3955)			(−1.4163)		(−1.6228)
*Constant*	−0.6285	−4.1160	16.5471	−0.0699	−3.7714	−1.7359	−4.9795
	(−0.0982)	(−0.8716)	(1.0131)	(−0.0106)	(−0.7907)	(−0.2496)	(−0.9787)
*Industry fixed effects*	Yes	Yes	Yes	Yes	Yes	Yes	Yes
*Year fixed effects*	Yes	Yes	Yes	Yes	Yes	Yes	Yes
*Observations*	6,340	6,340	7,503	6,594	6,594	6,402	6,402
*R-squared*	0.0966	0.1200	0.0480	0.0938	0.1182	0.0948	0.1204

(1) Robust standard errors are clustered by industry and t-statistics are reported in parentheses; (2) *, **, *** represent significant at the 10, 5, and 1% significance level, respectively.

### Short-Term and Long-Term Effect

We divide the post-policy period into three segments: (1) 1 year after the implementation of GCP; (2) 2 years after the implementation of GCP; and (3) 3 years after the implementation of GCP. In this way, we estimate the short-term and long-term impact of GCP on stock price crash risk. The regression results in Table 6 shows that the policy has the most pronounced impact on the stock price crash risk of heavy-polluting firms 1 year after its implementation than 2 and 3 years after, that is, GCP has a more pronounced impact on stock price crash risk in the short term. First, the implementation of the GCP is an exogenous event that firms cannot control, leaving them unprepared with specialized risk mitigation measures. So, the short-term policy impact is stronger than the long-term one. Second, firms will gradually engage in green innovation, increase environmental expenditures, and seek to transform into an energy-saving, green, and low-carbon production model in the long run, so as to mitigate the policy impacts (Goetz, 2019; He et al., 2019).

**TABLE 6:** Short-term and long-term effect.

	One year after the implementation of GCP		Two years after the implementation of GCP		Three years after the implementation of GCP	
	(1)	(2)	(3)	(4)	(5)	(6)
Variable	*NCSKEW*_*t+1*_	*DUVOL*_*t+1*_	*NCSKEW*_*t+1*_	*DUVOL*_*t+1*_	*NCSKEW*_*t+1*_	*DUVOL*_*t+1*_
*treat*_*i*_×*policy*_*t*_	0.5216***	0.4981***	0.1447	0.1970**	0.1612**	0.2073***
	(2.9640)	(2.7430)	(1.6638)	(2.3529)	(2.1766)	(3.4412)
*policy*_*t*_	−0.3121***	−0.1396*	0.3945***	0.4751***	−0.1765**	−0.1280**
	(−3.6748)	(−1.9776)	(4.0380)	(4.8468)	(−2.2878)	(−2.0353)
*treat*_*i*_	0.2880***	0.2042**	0.0591	0.0318	−0.0868	−0.0891**
	(3.1160)	(2.1967)	(1.0278)	(0.5737)	(−1.6214)	(−2.1215)
*Lev*_*t*_	−0.0842	−0.1480	−0.2162*	−0.2454***	−0.1344	−0.2079***
	(−0.6874)	(−1.6607)	(−1.8917)	(−2.8368)	(−1.3597)	(−2.7404)
*ROE*_*t*_	0.3088	−0.0874	0.5983***	0.2066	0.4487***	0.1567
	(1.5886)	(−0.5291)	(3.0610)	(1.4252)	(2.6802)	(1.2984)
*Cashflow*_*t*_	−0.4322	−0.3291	−0.3561	−0.2485	−0.3931*	−0.2946
	(−1.3715)	(−1.1346)	(−1.2328)	(−1.0532)	(−1.6791)	(−1.4522)
*Balance*_*t*_	0.0244	0.0034	−0.0027	−0.0090	0.0027	−0.0023
	(0.5554)	(0.1129)	(−0.0804)	(−0.3510)	(0.1041)	(−0.1038)
*Dturn*_*t*_	−0.1695**	−0.1809***	−0.1485**	−0.1436**	−0.0778	−0.0982*
	(−2.5429)	(−3.0765)	(−2.1205)	(−2.3971)	(−1.2257)	(−1.8077)
*age*_*t*_	0.0053	0.0043	0.0091*	0.0073**	0.0029	0.0028
	(0.9759)	(1.0675)	(1.9339)	(2.0239)	(0.7789)	(0.9420)
*lnsize*_*t*_	1.5854***	1.8335***	−0.2904	0.2512	−0.2670	0.3631
	(4.4829)	(5.5284)	(−0.6948)	(0.9118)	(−0.7137)	(1.4654)
*NCSKEW*_*t*_	0.0439**		0.0374*		0.0259*	
	(2.1977)		(1.9394)		(1.7213)	
*Sigma*_*t*_	6.8395***	5.0624***	6.5047***	4.9866***	5.1500***	3.8657***
	(3.4901)	(2.9162)	(4.4123)	(3.8358)	(3.6963)	(3.4086)
*Ret*	0.0004	0.0028	0.0014	0.0031	−0.0038	−0.0014
	(0.1087)	(0.7960)	(0.3295)	(0.8490)	(−0.9966)	(−0.4118)
*TobinQ*	0.0804***	0.0651***	0.0476**	0.0427***	0.0442**	0.0396***
	(2.8670)	(2.8433)	(2.2367)	(2.6810)	(2.5672)	(3.0346)
*DUVOL*		0.0021		−0.0098		−0.0210
		(0.1132)		(−0.5175)		(−1.4018)
Constant	−16.3681	−15.1529*	−17.9883*	−16.0087**	−5.5492	−7.1647
	(−1.5067)	(−1.8207)	(−1.9084)	(−2.2900)	(−0.7389)	(−1.2477)
*Industry fixed effects*	Yes	Yes	Yes	Yes	Yes	Yes
*Year fixed effects*	Yes	Yes	Yes	Yes	Yes	Yes
*Observations*	3,188	3,188	4,399	4,399	5,515	5,515
*R-squared*	0.1076	0.1171	0.1225	0.1343	0.1100	0.1327

(1) Robust standard errors are clustered by industry and t-statistics are reported in parentheses; (2) *, **, *** represent significant at the 10, 5, and 1% significance level, respectively.

### Alternative Measures

Following Kim et al. (2016), we use a dummy variable *CRASH* as an alternative measure of stock price crash risk to re-estimate the baseline regression, and the results are presented in column (3) of Table 5. The results show that the coefficient of *treat*_*i*_×*policy*_*t*_ is significantly positive at the 1% significance level and the regression results remain robust.

### Other Robustness Checks

According to the *Key Performance Indicators for Implementing Green Credit*, Category B industries may also have adverse environmental and social consequences in addition to Category A industries. Following Wang and Wang (2021), we include firms in the Category B industries^8^ in the treatment group. As seen from columns (4) and (5) in Table 5, the coefficients of *treat*_*i*_×*policy*_*t*_ are still significantly positive. Considering the important influence of managers on corporate operation, we further control for CEO-level characteristics, such as whether the CEO also serves as the chairman of the board (*IsDuality*_*i*_), CEO gender (*gender*_*i*_), and whether the CEO has an overseas background (*oveseaback*_*i*_). The regression results are presented in columns (6) and (7) of Table 5. It shows that the coefficients of *treat*_*i*_×*policy*_*t*_ remain significant, indicating that GCP can significantly increase the price crash risk of heavy-polluting firms.

### Heterogeneity Analysis

#### External Corporate Governance

Following An and Zhang (2013), we use the shareholding ratio of institutional investors to measure external corporate governance quality in order to investigate if it affects the relationship between GCP and the stock price crash risk of heavy-polluting firms. If a firm’s shareholding ratio of institutional investors is higher than the mean value of the sample firms, the firm’s external governance is deemed high-quality, while the opposite is considered low-quality.

As shown in Table 7, the coefficients of *treat*_*i*_×*policy*_*t*_ are significant at the 5 and 1% level in the group with low-quality external governance, but not in the group with high-quality external governance. This indicates that GCP increases the stock price crash risk of heavy-polluting firms with weak external governance significantly more than those with adequate external governance, which proves Hypothesis 2.

**TABLE 7:** Impact of external corporate governance.

	Low-quality external governance		High-quality external governance	
Variable	*NCSKEW*_*t+1*_	*DUVOL*_*t+1*_	*NCSKEW*_*t+1*_	*DUVOL*_*t+1*_
*treat*_*i*_×*policy*_*t*_	0.2355**	0.2853***	0.0700	0.1063
	(2.4950)	(3.8107)	(0.9392)	(1.6002)
*policy*_*t*_	−0.0540	0.0357	−0.0503	0.0423
	(−0.6470)	(0.4558)	(−0.5394)	(0.5420)
*treat*_*i*_	−0.1833***	−0.3231***	−0.0588	0.0105
	(−2.6642)	(−5.9630)	(−1.0895)	(0.2287)
*Lev*_*t*_	−0.1664	−0.1915**	0.0314	−0.1004
	(−1.4321)	(−2.2901)	(0.3311)	(−1.4938)
*ROE*_*t*_	0.2520	0.0517	0.4970***	0.2476*
	(1.4424)	(0.3906)	(2.9833)	(1.9922)
*Cashflow*_*t*_	−0.5588**	−0.3828**	−0.1395	−0.1274
	(−2.5895)	(−2.0663)	(−0.4329)	(−0.5000)
*Balance*_*t*_	−0.0076	−0.0147	0.0294	0.0165
	(−0.2999)	(−0.6445)	(0.7779)	(0.5785)
*Dturn*_*t*_	−0.1658**	−0.1702***	0.0111	−0.0148
	(−2.6011)	(−3.1893)	(0.1188)	(−0.1922)
*age*_*t*_	−0.0019	0.0009	0.0027	0.0016
	(−0.4425)	(0.2836)	(0.6645)	(0.5751)
*lnsize*_*t*_	−0.1388	0.4646	−0.6694*	0.0116
	(−0.2960)	(1.3039)	(−1.7123)	(0.0400)
*NCSKEW*_*t*_	0.0170		0.0185	
	(1.0546)		(1.0933)	
*Sigma*_*t*_	4.6793**	4.2238***	3.7981***	2.9188***
	(2.5419)	(2.8033)	(3.5156)	(3.1974)
*Ret*_*t*_	0.0021	0.0046	−0.0108***	−0.0075***
	(0.4007)	(1.0775)	(−3.9416)	(−2.9180)
*TobinQ*_*t*_	0.0021	0.0086	0.0157	0.0216
	(0.1267)	(0.6414)	(0.7906)	(1.4845)
*DUVOL*_*t*_		−0.0129		−0.0285*
		(−0.6433)		(−1.8271)
*Constant*	3.8904	−3.5619	−3.7304	−3.8573
	(0.4528)	(−0.5439)	(−0.4656)	(−0.6839)
*Industry fixed effects*	Yes	Yes	Yes	Yes
*Year fixed effects*	Yes	Yes	Yes	Yes
*Observations*	3,118	3,118	3,459	3,459
*R-squared*	0.1188	0.1556	0.1010	0.1123

(1) Robust standard errors are clustered by industry and t-statistics are reported in parentheses; (2) *, **, *** represent significant at the 10, 5, and 1% significance level, respectively.

#### Firm Size

To investigate whether the relationship between GCP and the stock price crash risk of heavy-polluting firms is affected by firm size, we divide the sample into subgroups according to the following criteria and regress the subgroups: if a firm’s market value^9^ exceeds the sample’s mean value (Cui et al., 2021), it is classified as a large-sized firm; otherwise, a small-sized firm.

Table 8 reports the regression results by firm size, which show that the coefficients of *treat*_*i*_×*policy*_*t*_ for the small-sized firms are significantly positive at the 1% level, but are insignificant for the large-sized firms. It can be concluded that the impact of GCP on the stock price crash risk of heavy-polluting firms is more pronounced in small-sized firms. This supports Hypothesis 3.

**TABLE 8:** Impact of firm size.

	Small-sized firm		Large-sized firm	
Variable	*NCSKEW*_*t+1*_	*DUVOL*_*t+1*_	*NCSKEW*_*t+1*_	*DUVOL*_*t+1*_
*treat*_*i*_×*policy*_*t*_	0.2449***	0.2772***	0.2103	0.2218
	(2.7448)	(3.6809)	(0.9233)	(1.2842)
*policy*_*t*_	−0.1000	0.0277	−0.1170	−0.1410
	(−1.5017)	(0.4643)	(−0.7422)	(−0.8593)
*treat*_*i*_	−0.1091*	−0.1469***	−0.3282*	−0.0515
	(−1.7683)	(−2.7869)	(−1.9248)	(−0.4111)
*Lev*_*t*_	−0.0578	−0.1236*	−0.2623	−0.3174*
	(−0.6557)	(−1.9416)	(−1.1142)	(−1.9796)
*ROE*_*t*_	0.3106**	0.1056	0.5124*	0.2808
	(2.4325)	(1.2015)	(1.7230)	(1.2177)
*Cashflow*_*t*_	−0.3452	−0.2576	−0.2429	−0.1713
	(−1.5787)	(−1.4952)	(−0.5834)	(−0.5557)
*Balance*_*t*_	−0.0020	−0.0057	0.0352	0.0148
	(−0.0878)	(−0.3089)	(0.8628)	(0.3980)
*Dturn*_*t*_	−0.0778	−0.0859**	−0.1166	−0.1238
	(−1.4947)	(−2.0295)	(−0.9198)	(−1.0435)
*age*_*t*_	0.0021	0.0025	0.0005	0.0005
	(0.6470)	(0.9501)	(0.0698)	(0.1279)
*lnsize*_*t*_	0.0787	0.5024	−2.2342*	−1.6789*
	(0.1651)	(1.4907)	(−1.8828)	(−1.8600)
*NCSKEW*_*t*_	0.0349***		−0.0062	
	(2.7688)		(−0.2428)	
*Sigma*_*t*_	3.8536***	3.4022***	5.8495***	3.9459**
	(3.4788)	(3.8216)	(2.8996)	(2.3024)
*Ret*_*t*_	−0.0027	−0.0006	−0.0159**	−0.0107
	(−0.7468)	(−0.1824)	(−2.3154)	(−1.6044)
*TobinQ*_*t*_	0.0208	0.0198*	−0.0266	−0.0121
	(1.4010)	(1.7727)	(−0.9962)	(−0.4847)
*DUVOL*_*t*_		0.0001		−0.0421
		(0.0041)		(−1.2342)
*Constant*	−4.8440	−7.0050	5.6454	3.8478
	(−0.7969)	(−1.4005)	(0.4168)	(0.5357)
*Industry fixed effects*	Yes	Yes	Yes	Yes
*Year fixed effects*	Yes	Yes	Yes	Yes
*Observations*	5,324	5,324	1,270	1,270
*R-squared*	0.1182	0.1555	0.1246	0.1225

(1) Robust standard errors are clustered by industry and t-statistics are reported in parentheses; (2) *, **, *** represent significant at the 10, 5, and 1% significance level, respectively.

## Mediating Analysis

The baseline results in section “The Impact of Green Credit Policy on Stock Price Crash Risk” demonstrate that green credit policy (GCP) raises the stock price crash risk of heavy-polluting firms significantly. In this section, we will further examine how corporate information transparency and financial constraints play a role in transferring the impact of GCP on stock price crash risk.

### Financial Constraints

Following Hadlock and Pierce (2010), we use sa_*t* + 1_^10^ to measure corporate financial constraints. Table 9 reports the results for the mediating effect of financial constraints. As shown in column (1), the impact of GCP on the stock price crash risk of heavy-polluting firms is positively transmitted by the firms’ financial constraints, indicating that GCP increases the financial distress of heavy-polluting firms. In addition, the results in columns (2) and (3) indicate that the increased financial constraints caused by GCP on heavy-polluting firms results in significant increase in their price crash risk.

**TABLE 9:** Impact of corporate financial constraints.

	(1)	(2)	(3)
Variable	sa_*t* + 1_	*NCSKEW*_*t+1*_	*DUVOL*_*t+1*_
*treat*_*i*_×*policy*_*t*_	0.0007**	0.1526**	0.1987***
	(2.3639)	(2.0362)	(3.2803)
sa_*t* + 1_		11.4387***	11.5029***
		(5.8249)	(6.5950)
*policy*_*t*_	0.0013***	−0.1909**	−0.1424**
	(4.9234)	(−2.5165)	(−2.3170)
*treat*_*i*_	−0.0038***	−0.0429	−0.0449
	(−16.6890)	(−0.8089)	(−1.0807)
*Lev*_*t*_	−0.0013***	−0.1191	−0.1924**
	(−2.6959)	(−1.1957)	(−2.5078)
*ROE*_*t*_	−0.0074***	0.5339***	0.2425*
	(−7.1830)	(3.1298)	(1.9405)
*Cashflow*_*t*_	0.0049***	−0.4491**	−0.3510*
	(2.7270)	(−1.9988)	(−1.7925)
*Balance*_*t*_	−0.0001	0.0038	−0.0011
	(−0.9841)	(0.1488)	(−0.0499)
*Dturn*_*t*_	0.0002	−0.0798	−0.1002*
	(0.6878)	(−1.2558)	(−1.8528)
*age*_*t*_	−0.0001***	0.0038	0.0036
	(−4.8696)	(1.0173)	(1.2601)
*lnsize*_*t*_	−0.4630***	5.0287***	5.6883***
	(−197.3469)	(4.9284)	(6.6290)
*NCSKEW*_*t*_	−0.0001	0.0266*	
	(−0.4538)	(1.8169)	
*DUVOL*_*t*_	0.0000		−0.0202
	(0.1160)		(−1.3726)
*Sigma*_*t*_	0.0042	5.1029***	3.8162***
	(0.5118)	(3.5860)	(3.3112)
*Ret*_*t*_	−0.0000	−0.0038	−0.0013
	(−0.0939)	(−0.9894)	(−0.4056)
*TobinQ*_*t*_	−0.0007***	0.0525***	0.0480***
	(−3.6765)	(3.1271)	(3.8260)
*Constant*	−0.5971***	1.2817	−0.2970
	(−18.8777)	(0.1651)	(−0.0486)
*Industry fixed effects*	Yes	Yes	Yes
*Year fixed effects*	Yes	Yes	Yes
*Observations*	5,515	5,515	5,515
*R-squared*	0.9680	0.1129	0.1370

(1) Robust standard errors are clustered by industry and t-statistics are reported in parentheses; (2) *, **, *** represent significant at the 10, 5, and 1% significance level, respectively.

### Corporate Information Transparency

Following Lang et al. (2012), we use analysts’ earnings forecast accuracy, *ACCURACY*_*t+1*_, to measure corporate information transparency; the higher the value of *ACCURACY*, the more information transparency the firm has. Table 10 reports the results for the mediating effect of corporate information transparency. As shown in column (1), the impact of GCP is negatively related to the information transparency of heavy-polluting firms, indicating that GCP decreases the information transparency of heavy-polluting firms. In addition, the results in columns (2) and (3) show that the negative impact of GCP on the information transparency of heavy-polluting firms can increase their price crash risk.

**TABLE 10:** Impact of corporate information transparency.

	(1)	(2)	(3)
Variable	*ACCURACY*_*t+1*_	*NCSKEW*_*t+1*_	*DUVOL*_*t+1*_
*treat*_*i*_×*policy*_*t*_	−0.0096**	0.1711**	0.1960***
	(−2.1193)	(2.2912)	(3.2310)
*ACCURACY*_1*t* + 1_		−1.2674***	−1.4484***
		(−4.2371)	(−5.8661)
*policy*_*t*_	−0.0027	−0.1795**	−0.1238*
	(−0.7345)	(−2.1520)	(−1.8200)
*treat*_*i*_	0.0301***	−0.0116	0.0108
	(10.1236)	(−0.2085)	(0.2496)
*Lev*_*t*_	−0.0170**	−0.1005	−0.1908**
	(−2.2872)	(−0.9057)	(−2.1076)
*ROE*_*t*_	0.0824***	0.4626**	0.1957
	(5.2669)	(2.2249)	(1.3375)
*Cashflow*_*t*_	0.0304**	−0.3596	−0.2405
	(2.3892)	(−1.4913)	(−1.1306)
*Balance*_*t*_	−0.0000	0.0040	−0.0054
	(−0.0219)	(0.1477)	(−0.2374)
*Dturn*_*t*_	−0.0013	−0.0805	−0.0976*
	(−0.4310)	(−1.2155)	(−1.7937)
*age*_*t*_	−0.0002	0.0024	0.0025
	(−0.9985)	(0.5964)	(0.7943)
*lnsize*_*t*_	−0.1339***	−0.4576	0.1370
	(−3.7859)	(−1.1251)	(0.5081)
*NCSKEW*_*t*_	0.0048**	0.0136	
	(2.0468)	(0.9551)	
*DUVOL*_*t*_	−0.0167***		−0.0398***
	(−6.2207)		(−2.7094)
*Sigma*_*t*_	−0.1833**	5.0573***	3.3163***
	(−2.2680)	(3.1535)	(2.6784)
*Ret*_*t*_	−0.0001	−0.0037	−0.0010
	(−0.7849)	(−0.9424)	(−0.3066)
*TobinQ*_*t*_	0.0025***	0.0552***	0.0497***
	(3.4180)	(3.1043)	(3.5267)
*Constant*	0.7319**	−4.0866	−5.9671
	(2.1641)	(−0.4820)	(−0.9454)
*Industry fixed effects*	Yes	Yes	Yes
*Year fixed effects*	Yes	Yes	Yes
*Observations*	4,986	4,986	4,986
*R-squared*	0.2154	0.1128	0.1381

(1) Robust standard errors are clustered by industry and t-statistics are reported in parentheses; (2) *, **, *** represent significant at the 10, 5, and 1% significance level, respectively.

## Conclusion and Policy Implications

This paper explores the impact of green credit policy (GCP) on stock price crash risk using Chinese A-share listed firms as the research setting in the context of the *Green credit guidelines* issued by the China Banking Regulatory Commission (CBRC) in 2012. The DID results show that the implementation of GCP significantly increases stock price crash risk, and this finding passes a battery of robustness checks. However, the results are influenced by two factors: the quality of external governance and the size of the firm, and GCP has a more significant impact on the stock price crash risk in heavy-polluting firms with weak external governance and a small size. In addition, the mediating analysis shows that corporate information transparency and financial constraints play a mediating role in transferring the impact of GCP on stock price crash risk. The GCP increases the financial constraints of heavy-polluting firms and decreases their information transparency, which in turn affects their risk of a price crash.

The implementation of GCP increases the risk of heavy-polluting firms experiencing price crashes, prompting them to invest in green projects, minimize negative environmental impacts, and enhance corporate sustainability. Based on our findings, we propose the following policy recommendations to strengthen GCP’s environmental regulatory effect: First, commercial banks, as the examination and approval department in the execution of GCP, should strictly adhere to GCP regulations when granting loans to firms, and higher authorities should provide proper oversight and inspection. Second, GCP’s goal is not to trigger a stock market crash but to rationalize capital allocation. Therefore, in order to reduce the price crash risk, heavy-polluting firms should enhance corporate information transparency and expand corporate financing channels by strengthening corporate compliance, improving external governance, and increasing firm value. Finally, green transformation is the key to corporate sustainability. Heavy-polluting firms should enhance their environmental awareness, expedite green transformation and innovation, and lower corporate risks to achieve corporate sustainable development and fulfill social and environmental responsibility.

## Data Availability Statement

Publicly available datasets were analyzed in this study. This data can be found here: https://www.gtarsc.com and https://www.wind.com.cn.

## Author Contributions

WZ, YL, FZ, and HD contributed to conception and design of the study. WZ and YL organized the database and performed the statistical analysis. All authors wrote the first draft of the manuscript, contributed to manuscript revision, read, and approved the submitted version.

## Conflict of Interest

The authors declare that the research was conducted in the absence of any commercial or financial relationships that could be construed as a potential conflict of interest.

## Publisher’s Note

All claims expressed in this article are solely those of the authors and do not necessarily represent those of their affiliated organizations, or those of the publisher, the editors and the reviewers. Any product that may be evaluated in this article, or claim that may be made by its manufacturer, is not guaranteed or endorsed by the publisher.

## Footnotes

1 According to the document “*Key Performance Indicators for Implementing Green Credit*,” Class A industries include: nuclear power generation; hydro power generation; water conservancy and inland river port construction; coal mining and washing; oil and gas extraction; ferrous metal mining and processing; non-ferrous metal mining and processing; non-metallic mining and processing; other mining industries.

2 In 1973, the State Council held the first National Working Conference on Ecological and Environmental Protection; in 1983, the second National Working Conference on Ecological and Environmental Protection established environmental protection as a basic state policy; in 1984, the *Decision on Environmental Protection* was issued; in 1988, the State Environmental Protection Administration was established; in 1979, the *Environmental Protection Law of the People’s Republic of China* was trialed and went into law in 1989; in 1992, *China’s Ten Strategic Policies on Environment and Development* was announced; in 1996, the *Decision on Several Issues of Environmental Protection* was issued; in 1994, *China’s Agenda 21 was* promulgated; in 2019, the government work report states that it will continue to promote pollution prevention and control, and consolidate and expand the achievements of the Blue Sky Defense War, while specifying that SO_2_ and NOx emissions will fall by 3% and PM2.5 concentrations will continue to fall in key areas; in February 2021, the State Council issued the *Guiding Opinions on Accelerating the Establishment and Improvement of a Green and Low-Carbon Circular Development Economic System*, which states that “establishing and improving a green and low-carbon circular development economic system and promoting a comprehensive green transformation of economic and social development are the basic policies for solving China’s resource, environmental, and ecological problems.”

3 The data was retrieved from the *2019 Bulletin of Ecology and Environment Status of China* http://www.mee.gov.cn/hjzl/sthjzk/zghjzkgb/.

4
http:/www.pbc.gov.cn

5 Category A includes: nuclear power generation; hydro power generation; water conservancy and inland river port construction; coal mining and washing; oil and gas extraction; ferrous metal mining and processing; non-ferrous metal mining and processing; non-metallic mining and processing; other mining industries.

6
https://www.wind.com.cn/

7
https://www.gtarsc.com/

8 According to the *Key Performance Indicators for Implementing Green Credit*, Category B includes 25 types of industries, including cotton dyeing, printing, and finishing; wool dyeing and finishing; hemp dyeing and finishing; silk dyeing, printing, and finishing; chemical fiber dyeing, printing, and finishing; knitting or crochet dyeing, printing, and finishing; leather tanning and finishing; fur tanning and finishing; pulp manufacturing; paper making; petroleum refining, coking and nuclear fuel processing; chemical material and product manufacturing; pharmaceutical manufacturing; rubber and plastic products; non-metallic mineral products; ferrous metal smelting and rolling processing; non-ferrous metal smelting and rolling processing; thermal power generation; heat power generation and supply; gas generation and supply; house building; civil engineering construction; railroad transportation; intra-city and inter-city rail-transit; pipeline transport.

9 This data comes from the China Stock Market Accounting Research (CSMAR).

10 According to Hadlock and Pierce (2010), sa_*t* + 1_ = −0.737 * *size*_*it+1*_ + 0.043 * $s\,i\,z\,e_{i\,t+1}^{2}$−0.04 * *age*_*it+1*_, where *size*_*it+1*_ denotes firm size and *age*_*it+1*_ represents firm age.

## Appendix A

**Appendix A:** Variable definitions.

Variables	Definitions
*NCSKEW*_*t+1*_	Skewness of the negative stock return
*DUVOL*_*t+1*_	Fluctuation in the stock return.
*treat*_*i*_	An indicator variable takes the value of 1 if firm *i* belongs to the treatment group (i.e., the firm belongs to heavy-polluting industries), and 0 otherwise.
*policy*_*t*_	A binary variable takes the value of 1 after the green credit policy (2015–2012), and 0 otherwise (2009–2011).
*Lev*_*t*_	The book value of total debt divided by the book value of total assets.
*ROE*_*t*_	The net profit divided by the average balance of shareholder equity
*Cashflow*_*t*_	Net cash flow from operating activities divided by total assets
*Balance*_*t*_	The sum of the shareholdings of the second to fifth largest shareholders divided by the shareholding of the first largest shareholder
*Dturn*_*t*_	the average monthly share turnover in year t minus the average monthly share turnover in t –1
*age*_*t*_	A firm age since listing
*lnsize*_*t*_	Natural logarithm of a firm’s total assets
*Sigma*_*t*_	Standard deviation of the stock’s firm-specific weekly return
*Ret*_*t*_	Mean of the stock’s firm-specific weekly return.
*TobinQ*_*t*_	The net profit divided by the average balance of shareholder equity
*IsDuality*_*i*_	Whether the company’s chairman and CEO are the same person
*gender*_*i*_	A dummy variable takes the value of 1 if the CEO is male and 0 if female
*oveseaback*_*i*_	A dummy variable takes the value of 1 if the CEO has overseas background, 0 otherwise
